# Multidimensional biomarker profiling of bronchoalveolar lavage fluid for diagnostic and prognostic evaluation of lung cancer: a retrospective observational study

**DOI:** 10.3389/fmed.2026.1774382

**Published:** 2026-03-27

**Authors:** Yu Wang, Jie Huang, Xiaomeng Xie, Shaolin Li, Qiang Fu

**Affiliations:** 1 Department of Respiratory, The Fifth Affiliated Hospital of Jinan University (Heyuan Shenhe People's Hospital), Heyuan City, Guangdong Province, China; 2 Department of Oncology, The Fifth Affiliated Hospital of Jinan University (Heyuan Shenhe People's Hospital), Heyuan City, Guangdong Province, China; 3 Department of Digestive, The Fifth Affiliated Hospital of Jinan University (Heyuan Shenhe People's Hospital), Heyuan City, Guangdong Province, China; 4 Department of Cardiovascular, The Fifth Affiliated Hospital of Jinan University (Heyuan Shenhe People's Hospital), Heyuan City, Guangdong Province, China; 5 Department of Urology, The First People's Hospital of Xiangcheng, Zhoukou City, Henan Province, China

**Keywords:** biomarkers, bronchoalveolar lavage fluid, circulating tumor DNA, cytokines, liquid biopsy, lung neoplasms, tumor microenvironment

## Abstract

**Background:**

Lung cancer remains a leading cause of cancer-related mortality worldwide. This study evaluated multidimensional biomarkers in bronchoalveolar lavage fluid (BALF) for the diagnosis and prognostic evaluation of lung cancer.

**Methods:**

This retrospective observational study included 100 patients who underwent clinically indicated bronchoscopy between April 2022 and April 2024, comprising 65 patients with histopathologically confirmed lung cancer and 35 patients with benign pulmonary diseases. BALF data obtained during routine clinical care were retrospectively collected for tumor markers (CEA, CYFRA21-1, NSE, ProGRP), cytokines (IL-6, IL-8, TNF-*α*, IL-10), ctDNA mutation profiles, and immune-cell subpopulations. Diagnostic performance was assessed using receiver operating characteristic (ROC) analysis. Overall survival (OS) was analyzed using Kaplan–Meier methods and Cox proportional hazards regression.

**Results:**

BALF concentrations of CEA, CYFRA21-1, NSE, ProGRP, IL-6, IL-8, and TNF-*α* were significantly higher in lung cancer patients than in benign controls, whereas IL-10 was lower, resulting in a markedly elevated IL-6/IL-10 ratio (all *p* < 0.05). ctDNA was detected more frequently in BALF than in peripheral blood (87.7% vs. 64.6%, *p* = 0.002), with higher variant allele frequencies and greater concordance with tissue genotyping (86.8%). Immune profiling showed increased regulatory T-cell proportions and M2 macrophage polarization with reduced CD8^+^ T-cell proportions in lung cancer BALF. In multivariable Cox analysis, advanced TNM stage, IL-6/IL-10 ratio >15, and CD8^+^/Treg ratio <1.5 were independent predictors of poorer OS.

**Conclusion:**

BALF provides tumor-proximal diagnostic and prognostic information in lung cancer. Enriched tumor markers, inflammatory imbalance, particularly the IL-6/IL-10 ratio, enhanced ctDNA detection, and local immunosuppressive immune features support the utility of BALF as a minimally invasive liquid biopsy for lung cancer evaluation and risk stratification.

## Introduction

Lung cancer is one of the most prevalent malignant tumors worldwide and remains a leading cause of cancer-related mortality (1). Despite continuous advances in diagnostic techniques and therapeutic strategies, the overall 5-year survival rate remains unsatisfactory, largely because the majority of patients are diagnosed at advanced stages of disease (2). Consequently, the identification of highly sensitive and specific diagnostic approaches, together with robust prognostic biomarkers, is of critical clinical importance for improving patient outcomes. Currently, lung cancer diagnosis relies primarily on imaging modalities and histopathological confirmation, including computed tomography (CT), positron emission tomography–computed tomography (PET-CT), and tissue biopsies obtained via percutaneous lung puncture or bronchoscopy (3). However, these methods have notable limitations. Imaging techniques may lack sufficient sensitivity for detecting small or early-stage lesions, while tissue biopsies are invasive and susceptible to tumor heterogeneity and sampling bias. These constraints highlight the urgent need for minimally invasive diagnostic strategies capable of providing accurate and comprehensive disease characterization. Bronchoalveolar lavage fluid (BALF), which directly reflects the pulmonary microenvironment, has attracted increasing attention as a diagnostic specimen for various lung diseases (4). BALF contains a wide range of cellular and acellular components, including proteins, nucleic acids, cytokines, and immune cells, that mirror local pathological and immunological alterations. Compared with peripheral blood, BALF sampling offers the advantage of proximity to the lesion, potentially enabling more sensitive detection of tumor-associated signals. Nevertheless, systematic investigations of BALF-derived biomarkers for lung cancer diagnosis and prognostic evaluation remain limited.

With the rapid development of liquid biopsy technologies, emerging approaches such as circulating tumor DNA (ctDNA) analysis, exosomal microRNA profiling, and immune-cell subpopulation assessment have opened new avenues for cancer detection and monitoring (5, 6). Applying these advanced techniques to BALF may further enhance diagnostic accuracy and deepen understanding of the tumor microenvironment. In parallel, the diagnostic and prognostic relevance of conventional tumor markers, including carcinoembryonic antigen (CEA), cytokeratin 19 fragment (CYFRA21-1), neuron-specific enolase (NSE), and pro-gastrin-releasing peptide (ProGRP), in BALF has not yet been fully elucidated.

Therefore, the present study aimed to systematically evaluate a panel of diagnostic and prognostic biomarkers in bronchoalveolar lavage fluid (BALF) from patients with lung cancer and benign pulmonary diseases. By assessing tumor markers, inflammatory cytokines, circulating tumor DNA, and immune-cell characteristics in BALF, this study sought to provide clinical evidence supporting the utility of BALF as a minimally invasive liquid biopsy for lung cancer diagnosis, risk stratification, and outcome assessment.

## Materials and methods

### Ethics statement

This retrospective study was approved by the Ethics Committee of The Fifth Affiliated Hospital of Jinan University(Heyuan Shenhe People’s Hospital), Heyuan City, 517,400, Guangdong Province, China (Approval No. SYJS2022-07-02). The study protocol complied with the principles of the Declaration of Helsinki. Written informed consent for bronchoscopy and the use of anonymized clinical data for research purposes was obtained from all patients as part of routine clinical practice.

### Study design and patients

This was a single-center, retrospective observational study conducted at The Fifth Affiliated Hospital of Jinan University (Heyuan Shenhe People’s Hospital), Heyuan City, Guangdong Province, China, between April 2022 and April 2024. During the study period, a total of 116 patients underwent clinically indicated bronchoscopy and were retrospectively screened for eligibility. Among them, 76 patients were initially suspected of lung cancer and 40 patients were suspected of benign pulmonary diseases based on clinical and radiological findings.

In the suspected lung cancer group, 11 patients were excluded due to incomplete clinical data (n = 8) or incomplete bronchoscopy procedures/insufficient sample collection (n = 3). The remaining 65 patients were histopathologically confirmed as lung cancer and were included in the final analysis. In the suspected benign pulmonary control group, 5 patients were excluded because of follow-up duration less than three months (n = 3) or diagnostic uncertainty (n = 2). Follow-up duration of at least three months was required for patients in the benign pulmonary control group. The remaining 35 patients were included as the benign pulmonary control group. Benign conditions included pulmonary infections, granulomatous diseases, and interstitial lung diseases. Ultimately, 100 patients (65 lung cancer patients and 35 patients in the benign pulmonary control group) constituted the final study population. As this was a retrospective study, no prospective sample size calculation was performed. The final sample size was determined by the number of eligible patients who met the inclusion criteria during the study period.

### Inclusion and exclusion criteria

Patients were eligible for inclusion if they were aged between 18 and 80 years; had pulmonary nodules or masses detected on chest computed tomography (CT) or positron emission tomography–computed tomography (PET-CT); underwent bronchoscopy for clinical diagnostic purposes; and had complete clinical, pathological, and laboratory data available for analysis. Patients were excluded if they had a prior history of lung cancer or other malignancies; had received chemotherapy, radiotherapy, targeted therapy, or immunotherapy within three months before bronchoscopy; had a severe pulmonary infection within one month prior to bronchoscopy; had significant cardiac, hepatic, or renal dysfunction, coagulation disorders, or other contraindications to bronchoscopy; were pregnant or lactating; or had cognitive impairment that interfered with procedural cooperation or follow-up; or, in the benign pulmonary disease group, had a follow-up duration of less than three months to exclude the possibility of occult malignancy. Severe pulmonary infection and significant cardiac, hepatic, or renal dysfunction were determined based on documented clinical diagnoses, laboratory test results, imaging findings, and treating physician assessments recorded in the electronic medical records at the time of bronchoscopy.

### Bronchoscopy and BALF collection

Information on bronchoscopy procedures and bronchoalveolar lavage fluid (BALF) collection was retrospectively obtained from medical and procedural records. All bronchoscopies had been performed by experienced respiratory physicians as part of routine clinical care using standard protocols, with an electronic bronchoscope (Olympus BF-1 T260 or equivalent). According to records, patients fasted prior to the procedure, and vital signs were monitored throughout. BALF was collected by instilling sterile saline (approximately 100 mL at 37 °C, administered in aliquots) into the bronchial subsegment corresponding to the lesion identified on imaging, followed by gentle aspiration. The recovered volume was documented in clinical records. BALF samples were processed in the clinical laboratory according to routine diagnostic procedures, including centrifugation for separation of supernatant and cellular components. When clinically indicated, bronchial brushings, mucosal biopsies, or transbronchial lung biopsies had been obtained for histopathological diagnosis.

### Tumor marker measurements

Concentrations of carcinoembryonic antigen (CEA), cytokeratin-19 fragment (CYFRA21-1), neuron-specific enolase (NSE), and pro-gastrin-releasing peptide (ProGRP) in BALF were retrospectively retrieved from clinical laboratory records. These measurements were performed as part of routine clinical testing using validated using electrochemiluminescent immunoassays performed on a Cobas e602 analyzer (Roche Diagnostics, Mannheim, Germany) according to the manufacturer’s standardized protocols.

### Cytokine measurements

Bronchoalveolar lavage fluid (BALF) cytokine concentrations were retrospectively obtained from clinical laboratory reports generated during routine diagnostic evaluation. The analyzed cytokines included interleukin-6 (IL-6), interleukin-8 (IL-8), tumor necrosis factor-*α* (TNF-α), and interleukin-10 (IL-10). Cytokine levels were measured using validated multiplex immunoassays according to the manufacturer’s instructions. The IL-6/IL-10 ratio was calculated to assess the balance between pro-inflammatory and anti-inflammatory signaling and was used in subsequent diagnostic and prognostic analyses.

### ctDNA analysis

circulating tumor DNA (ctDNA) results were retrospectively collected from molecular diagnostic reports generated during routine clinical care. ctDNA testing was performed on paired BALF supernatant and peripheral blood plasma samples using a validated targeted assay focused on four clinically relevant lung cancer genes (EGFR, TP53, KRAS, and ALK). For each specimen, mutation status, variant allele frequency (VAF), and ctDNA detection status were extracted from medical records. Concordance with tissue genotyping was defined as the presence of identical actionable alterations detected in both ctDNA and corresponding tissue testing (when tissue results were available).

### Immune cell subpopulation analysis

Immune cell subpopulation data in BALF were retrieved from routine clinical flow cytometry reports. Evaluated cell populations included CD8^+^ T cells, regulatory T cells (Tregs), macrophage subsets, and other immune cells as part of standard immunological assessment. Proportions of immune cell subsets and derived ratios were used for comparative and prognostic analyses.

### Pathological diagnosis

Pathological information was obtained retrospectively from existing cytology and histopathology reports. BALF cytology and bronchial biopsy findings had been generated at the time of clinical bronchoscopy as part of routine diagnostic care. All diagnoses used in this study were based solely on the original pathology reports issued by the hospital pathology department.

### Clinical data collection

Clinical data were extracted from electronic medical records, including age, sex, smoking history, clinical symptoms, imaging characteristics, histological subtype, and tumor stage. Tumor staging was performed according to the American Joint Committee on Cancer (AJCC) 8th edition TNM classification. Treatment information and follow-up outcomes were also recorded.

### Follow-up and outcome definition

Follow-up information was retrospectively obtained from medical records and hospital follow-up databases. Patients diagnosed with lung cancer had received standard treatment in accordance with prevailing clinical guidelines. Clinical assessments and imaging results recorded during routine follow-up were reviewed. Overall survival (OS) was defined as the interval from diagnosis to death from any cause.

### Statistical analysis

Statistical analyses were performed using SPSS version 26.0 (IBM Corp., Armonk, NY, USA) and R version 4.1.0 (R Foundation for Statistical Computing, Vienna, Austria). Descriptive statistics and group comparisons were conducted using SPSS as appropriate. Receiver operating characteristic (ROC) curve analysis, Kaplan–Meier survival curves, and Cox proportional hazards regression modeling were performed using R with the packages *pROC*, *survival*, and *survminer*. The proportional hazards assumption was evaluated using Schoenfeld residual testing, and no significant violations were observed. Continuous variables were expressed as mean ± standard deviation or median (interquartile range), depending on distribution. Categorical variables were presented as frequencies and percentages. The area under the ROC curve (AUC) with 95% confidence intervals (CIs) was calculated to assess diagnostic performance. Survival curves were compared using the log-rank test. Hazard ratios (HRs) with 95% CIs were estimated using Cox regression models. A two-sided *p* < 0.05 was considered statistically significant.

## Results

### Study population and baseline characteristics

**Figure 1:**
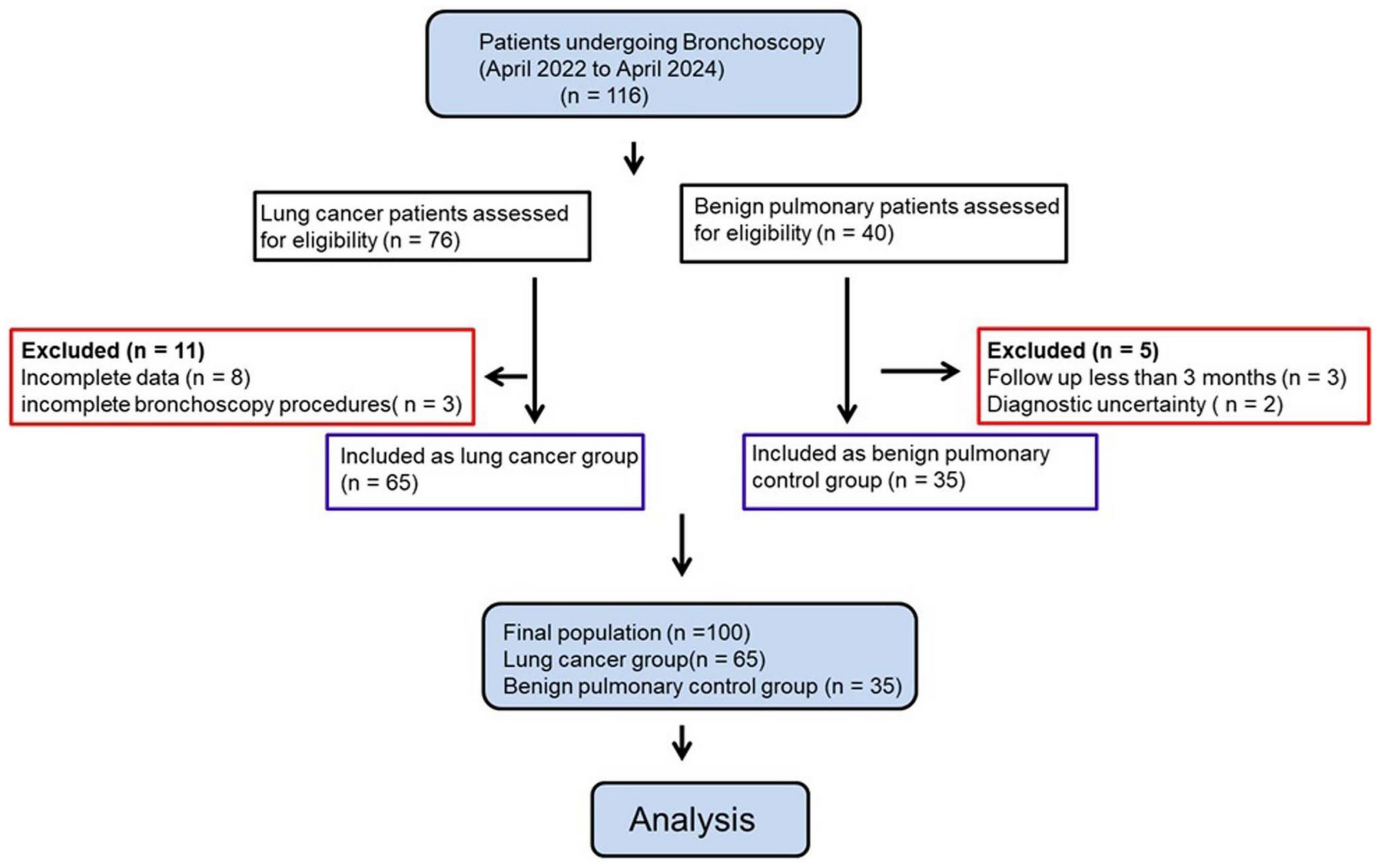
Study flow diagram. Flow diagram of patient screening, exclusion, and final inclusion in this retrospective study of BALF biomarkers in lung cancer.

Between April 2022 and April 2024, a total of 116 patients who underwent bronchoscopy were screened for eligibility. After exclusion of 11 lung cancer patients and 5 benign pulmonary control group patients based on predefined criteria, 100 patients were ultimately included in the analysis, comprising 65 patients with lung cancer and 35 patients with benign pulmonary disease (Figure 1). Baseline demographic and clinical characteristics are summarized in Table 1. There were no statistically significant differences between the lung cancer and benign pulmonary control group with respect to age or sex distribution. However, a significantly higher proportion of patients in the lung cancer group had a history of current or former smoking compared with the benign pulmonary control group (63.1% vs. 37.1%, *p* = 0.017). Among lung cancer patients, adenocarcinoma was the most common histological subtype (61.5%), followed by squamous cell carcinoma (27.7%), small cell lung cancer (7.7%), and other histologies (3.1%). Most lung cancer cases were diagnosed at advanced stages, with 38.5% classified as stage IV disease. The benign pulmonary control group consisted primarily of pulmonary infections, granulomatous diseases, and interstitial lung diseases (Table 1).

**Table 1:** Baseline characteristics of the study population.

Feature	Lung cancer group (*n* = 65)	Benign pulmonary control group (*n* = 35)	*p* value
Age (years), mean ± SD	63.7 ± 8.9	61.2 ± 10.4	0.206
Sex, *n* (%)			0.391
Male	42 (64.6)	20 (57.1)	
Female	23 (35.4)	15 (42.9)	
Smoking history, *n* (%)			0.017
Never smoker	24 (36.9)	22 (62.9)	
Current/former smoker	41 (63.1)	13 (37.1)	
Histological type, *n* (%)		–	–
Adenocarcinoma	40 (61.5)	–	
Squamous cell carcinoma	18 (27.7)	–	
Small cell lung cancer	5 (7.7)	–	
Other	2 (3.1)	–	
Clinical stage, *n* (%)		–	–
Stage I	11 (16.9)	–	
Stage II	8 (12.3)	–	
Stage III	21 (32.3)	–	
Stage IV	25 (38.5)	–	
Type of benign disease, *n* (%)	–		–
Pulmonary infection	–	20 (57.1)	
Granulomatous disease	–	8 (22.9)	
Interstitial lung disease	–	7 (20.0)	

### Tumor marker concentrations in BALF and peripheral blood

Comparisons of tumor marker concentrations between bronchoalveolar lavage fluid (BALF) and peripheral blood are presented in Table 2. In patients with lung cancer, BALF concentrations of CEA, CYFRA21-1, NSE, and ProGRP were all significantly higher than those observed in benign controls (all *p* < 0.001). Within the lung cancer group, BALF levels of CEA, CYFRA21-1, NSE, and ProGRP were significantly higher than corresponding peripheral blood levels (all p < 0.001), whereas differences between BALF and peripheral blood in the benign pulmonary control group were less pronounced. These findings indicate that BALF provides enhanced local enrichment of tumor-associated markers compared with peripheral circulation (Table 2).

**Table 2:** Comparison of tumor marker concentrations in BALF and peripheral blood.

Marker	Specimen	Lung cancer (*n* = 65)	Benign pulmonary control group (*n* = 35)	*P* value
CEA (ng/mL)	BALF	17.8 (9.6–42.7)	2.3 (1.4–3.8)	<0.001
	Peripheral blood	4.9 (2.8–12.5)	1.6 (0.9–2.4)	<0.001
	P (BALF vs. PB)	<0.001	0.037	
CYFRA21-1 (ng/mL)	BALF	14.6 (8.2–35.9)	3.1 (1.9–4.5)	<0.001
	Peripheral blood	5.3 (3.2–11.0)	1.8 (1.2–2.6)	<0.001
	P (BALF vs. PB)	<0.001	0.012	
NSE (ng/mL)	BALF	25.4 (14.9–56.3)	10.2 (6.8–15.4)	<0.001
	Peripheral blood	16.2 (10.5–23.7)	9.5 (7.1–12.3)	<0.001
	P (BALF vs. PB)	<0.001	0.214	
ProGRP (pg/mL)	BALF	72.8 (41.5–189.6)	27.4 (18.2–38.5)	<0.001
	Peripheral blood	46.2 (31.8–86.7)	25.1 (17.9–34.2)	<0.001
	P (BALF vs. PB)	<0.001	0.386	

### Inflammatory cytokine profiles in BALF and diagnostic performance

**Figure 2:**
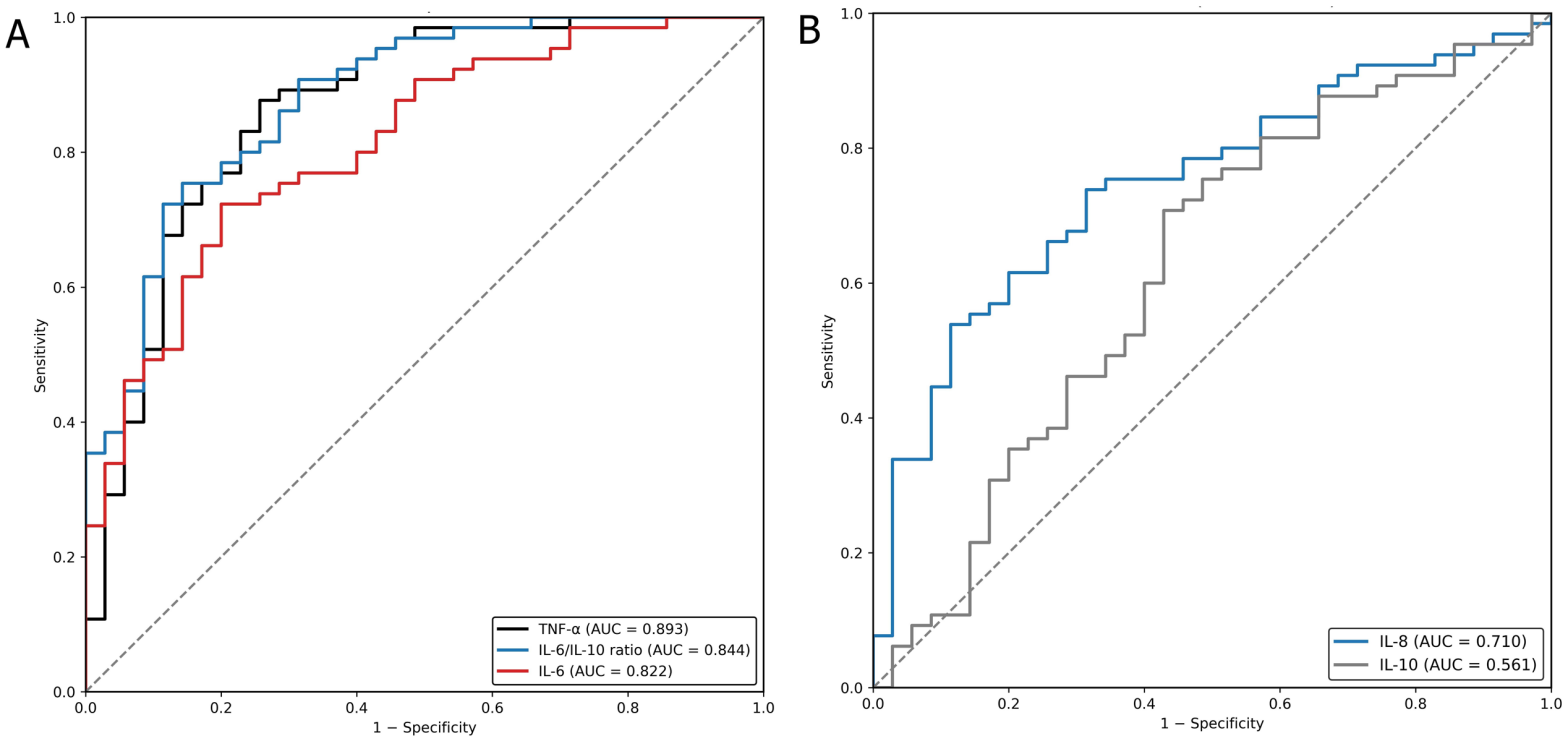
Diagnostic value of BALF cytokines. Receiver operating characteristic (ROC) curves showing the diagnostic performance of BALF cytokines for lung cancer. **(A)** IL-6, IL-10, and IL-6/IL-10 ratio. **(B)** IL-8 and TNF-*α*.

BALF inflammatory cytokine levels differed significantly between lung cancer patients and benign controls (Table 3). Lung cancer patients exhibited markedly elevated BALF concentrations of IL-6, IL-8, and TNF-*α*, along with significantly reduced IL-10 levels, resulting in a substantially higher IL-6/IL-10 ratio compared with benign controls (median 12.5 vs. 3.2, *p* < 0.001). Receiver operating characteristic (ROC) curve analysis demonstrated that TNF-*α* showed the highest diagnostic accuracy for lung cancer (AUC = 0.893), followed by the IL-6/IL-10 ratio (AUC = 0.844) and IL-6 (AUC = 0.822) (Figure 2A). Additional cytokines, including IL-8, and IL-10, displayed lower discriminatory performance (Figure 2B). These results indicate that specific BALF inflammatory markers, particularly TNF-α and the IL-6/IL-10 ratio, have strong diagnostic value for distinguishing lung cancer from benign pulmonary disease.

**Table 3:** Comparison of inflammatory factor levels in BALF.

Inflammatory factor	Lung cancer (*n* = 65)	benign pulmonary control group (*n* = 35)	*P* value
IL-6 (pg/mL)	85.6 (52.3–146.8)	31.7 (18.5–52.9)	<0.001
IL-8 (pg/mL)	215.3 (132.6–354.7)	74.2 (45.1–126.8)	<0.001
TNF-α (pg/mL)	18.7 (10.4–28.5)	9.8 (6.2–16.9)	0.002
IL-10 (pg/mL)	6.8 (4.2–10.5)	9.9 (6.4–15.7)	0.013
IL-6 / IL-10 ratio	12.5 (7.8–20.3)	3.2 (1.9–5.8)	<0.001

### ctDNA detection and mutational profiling in BALF versus peripheral blood

**Figure 3:**
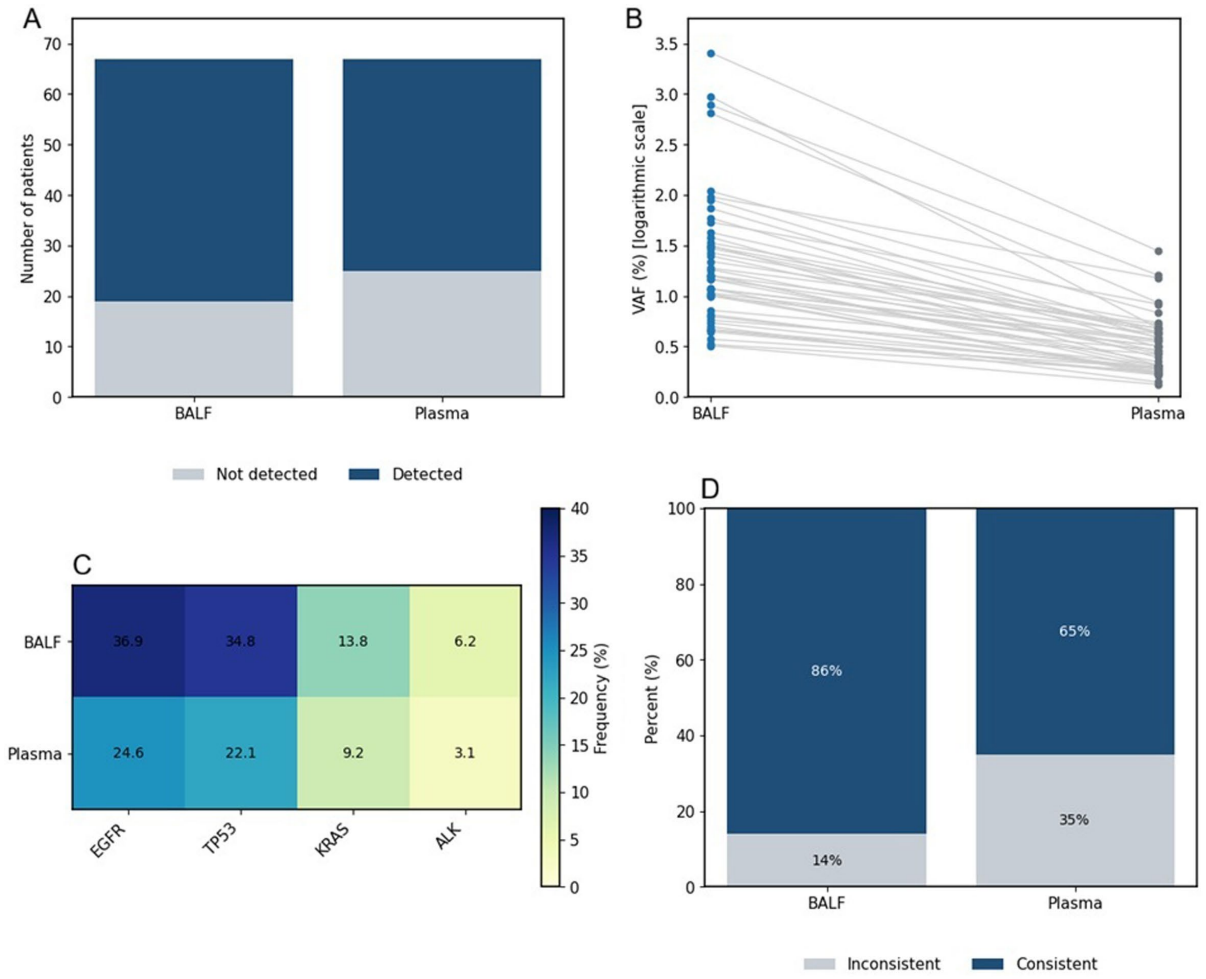
Comparison of ctDNA profiling in BALF and plasma. **(A)** ctDNA detection rates. **(B)** Paired variant allele frequencies (VAF). **(C)** Frequencies of major driver mutations. **(D)** Concordance with tissue genotyping.

Comparative analysis of circulating tumor DNA (ctDNA) detection in BALF and peripheral blood is shown in Figure 3 and Table 4. ctDNA was detected significantly more frequently in BALF than in peripheral blood among lung cancer patients (87.7% vs. 64.6%, *p* = 0.002). Furthermore, median variant allele frequency (VAF) was significantly higher in BALF compared with peripheral blood (4.72% vs. 1.70%, *p* < 0.001), as demonstrated by paired analyses (Figures 3A,B). Mutational profiling revealed that EGFR and TP53 were the most commonly detected alterations in BALF ctDNA, followed by KRAS and ALK (Figure 3C and Table 4). Concordance analysis with tissue genotyping showed that BALF ctDNA achieved a significantly higher concordance rate than peripheral blood ctDNA (86.8% vs. 67.9%, *p* = 0.020) (Figure 3D), underscoring the superior performance of BALF-derived ctDNA for molecular characterization.

**Table 4:** Comparison of BALF and peripheral blood ctDNA detection.

Feature	BALF	Peripheral blood	*P* value
ctDNA detection rate, n (%)	57 (87.7)	42 (64.6)	0.002
Variant allele frequency (%), median (range)	4.72 (0.72–32.5)	1.70 (0.51–15.2)	<0.001
EGFR mutation, n (%)	24 (36.9)	16 (24.6)	0.127
TP53 mutation, n (%)	22 (33.8)	15 (23.1)	0.168
KRAS mutation, n (%)	9 (13.8)	6 (9.2)	0.415
ALK rearrangement, n (%)	4 (6.2)	2 (3.1)	0.400
Concordance with tissue genotyping	46/53 (86.8)	36/53 (67.9)	0.020

### Prognostic significance of BALF biomarkers

The prognostic value of selected BALF biomarkers was evaluated using Cox proportional hazards regression analysis (Table 5). In univariate analyses, advanced TNM stage (III–IV), elevated IL-6/IL-10 ratio (>15), reduced CD8^+^/Treg ratio (<1.5), lower M1/M2 ratio (<0.5), and higher ctDNA VAF (>5%) were all significantly associated with poorer overall survival.

**Table 5:** Univariate and multivariate Cox regression analysis of overall survival.

Variable	Univariate HR (95% CI)	*P* value	Multivariate HR (95% CI)	*P* value
TNM stage (III–IV vs. I–II)	4.62 (1.73–12.35)	0.002	3.86 (1.37–10.89)	0.011
IL-6/IL-10 ratio (>15 vs. ≤ 15)	3.02 (1.51–6.04)	0.002	2.65 (1.28–5.47)	0.008
CD8^+^/Treg ratio (<1.5 vs. ≥ 1.5)	3.85 (1.79–8.29)	<0.001	3.18 (1.42–7.13)	0.005
M1/M2 ratio (<0.5 vs. ≥ 0.5)	2.87 (1.24–6.64)	0.014	–	–
ctDNA VAF (>5% vs. ≤ 5%)	2.64 (1.27–5.49)	0.009	–	–

**Figure 4:**
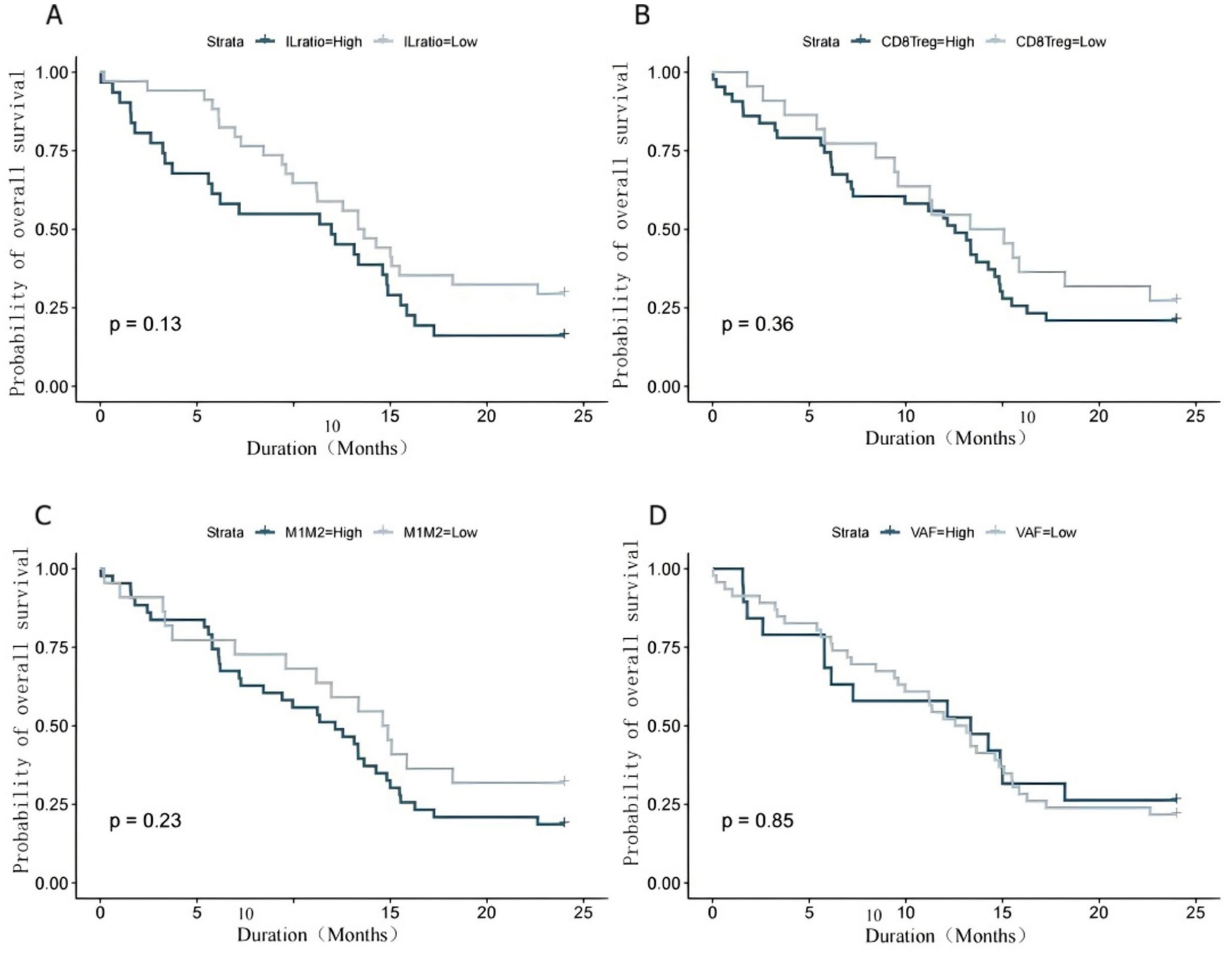
Survival analysis based on BALF biomarkers. Kaplan–Meier overall survival curves stratified by **(A)** IL-6/IL-10 ratio, **(B)** CD8^+^/Treg ratio, **(C)** M1/M2 macrophage ratio, and **(D)** ctDNA VAF.

In multivariate analysis, TNM stage III–IV (HR = 3.86, 95% CI 1.37–10.89, *p* = 0.011), IL-6/IL-10 ratio >15 (HR = 2.65, 95% CI 1.28–5.47, *p* = 0.008), and CD8^+^/Treg ratio <1.5 (HR = 3.18, 95% CI 1.42–7.13, *p* = 0.005) remained independent predictors of adverse overall survival (Table 5). Survival analyses demonstrated consistent trends toward reduced overall survival among patients with unfavorable biomarker profiles, including high IL-6/IL-10 ratio, low CD8^+^/Treg ratio, low M1/M2 ratio, and elevated ctDNA VAF, although some comparisons did not reach statistical significance (Figure 4).

### Immune cell alterations in BALF

Detailed immune cell profiling in BALF is provided in Supplementary Table S1. Compared with benign controls, lung cancer patients exhibited significantly reduced proportions of CD8^+^ T cells and markedly increased proportions of regulatory T cells (Tregs), resulting in a substantially lower CD8^+^/Treg ratio. Additionally, lung cancer BALF samples showed a pronounced shift toward an immunosuppressive macrophage phenotype, characterized by increased M2 macrophages and a reduced M1/M2 ratio. Elevated levels of myeloid-derived suppressor cells (MDSCs) were also observed. These findings indicate a distinctly immunosuppressive tumor microenvironment within the BALF of lung cancer patients (Supplementary Table S1).

## Discussion

This study provides comprehensive evidence that bronchoalveolar lavage fluid (BALF), as a tumor-proximal biological matrix, offers distinct advantages for lung cancer evaluation across diagnostic discrimination, molecular characterization, immune profiling, and prognostic stratification. We observed consistently higher concentrations of conventional tumor markers in BALF than in paired peripheral blood, as well as significantly higher levels in lung cancer compared with benign pulmonary disease, corroborating prior observations that airway-derived fluids concentrate disease-relevant proteins (7). Notably, BALF CEA and CYFRA21-1 demonstrated superior diagnostic performance compared with their peripheral counterparts, while ProGRP showed characteristic elevation in small cell lung cancer, in accordance with its established diagnostic specificity (8). These findings reinforce the concept that proximal airway sampling captures biologically enriched tumor signals that may be diluted in systemic circulation, as supported by studies showing that BALF is enriched for tumor-derived DNA compared with plasma and offers improved detection of cancer biomarkers compared with systemic blood sampling (9, 10). Inflammatory profiling further supported the biological relevance of BALF as a tumor-adjacent liquid biopsy. Lung cancer cases exhibited a distinct pro-inflammatory and immunosuppressive cytokine milieu characterized by elevated IL-6, IL-8, and TNF-*α* levels, reduced IL-10, and a markedly increased IL-6/IL-10 ratio. Importantly, the IL-6/IL-10 ratio outperformed individual cytokines and several conventional tumor markers for diagnostic discrimination in our cohort. This pattern aligns with the well-established roles of IL-6 in tumor proliferation, angiogenesis, and metastatic progression, alongside the complex immunomodulatory functions of IL-10 (11–13). Moreover, the association of the IL-6/IL-10 ratio with tumor burden and nodal involvement suggests that this composite index may integrate inflammatory drive with immune escape mechanisms. While serum-based IL-6/IL-10 ratios have previously been linked to tumor progression (13), our findings indicate that BALF accentuates these gradients, likely reflecting the immediate tumor microenvironment rather than systemic averages.

From a molecular perspective, BALF-derived circulating tumor DNA (ctDNA) demonstrated higher detection rates, greater variant allele frequencies, and stronger concordance with tissue genotyping compared with peripheral blood. These observations are consistent with emerging evidence that tumor-proximal fluids capture tumor-derived nucleic acids more efficiently than systemic samples (14–16). Of particular clinical relevance, actionable driver mutations were identified in BALF ctDNA in a subset of patients with negative tissue genotyping, potentially reflecting spatial tumor heterogeneity or sampling limitations inherent to tissue biopsies (15). The positive correlations between BALF ctDNA burden and both tumor size and TNM stage further support its utility as a surrogate marker of disease load, while its association with adverse survival outcomes underscores its prognostic relevance. Immune-cell profiling of BALF revealed a pronounced immunosuppressive signature, characterized by reduced CD8^+^ T-cell proportions, expansion of regulatory T cells (Tregs), a markedly decreased CD8^+^/Treg ratio, and polarization toward M2 macrophages. These features are hallmarks of a tumor microenvironment permissive to immune evasion and disease progression. Prior studies have demonstrated contact-dependent suppression of cytotoxic T-cell function by Tregs, consistent with our observations (17–20). Importantly, both the CD8^+^/Treg ratio and the M1/M2 macrophage ratio were associated with overall survival in our cohort, emphasizing that local immune balance within the airway microenvironment may exert prognostic influence beyond that captured by systemic immune indices.

By jointly evaluating tumor markers, inflammatory cytokines, ctDNA characteristics, and immune-cell parameters using conventional statistical and multivariable approaches, our study demonstrates that integrating complementary biological dimensions improves diagnostic discrimination and prognostic assessment compared with single-parameter analyses. The consistent contribution of the IL-6/IL-10 ratio, CEA, CYFRA21-1, ctDNA detection status, and immune-cell ratios highlights the value of combining tumor biology, inflammation, and immunity to better characterize lung cancer behavior (21). Notably, diagnostic performance remained robust in early-stage disease, a setting in which sensitivity is often limited yet clinically critical. Exploratory analyses further suggested that variation in BALF immune-inflammatory profiles may be associated with differences in clinical outcomes and treatment response. Concordance between BALF ctDNA and tissue genotyping correlated with better outcomes in patients receiving targeted therapy (22, 23). Driver mutation patterns detected in BALF mirrored established epidemiologic trends, including EGFR enrichment among non-smokers and women and KRAS predominance among smokers. Their prognostic associations were consistent with prior literature, with EGFR mutations generally conferring more favorable outcomes and TP53 mutations associated with poorer prognosis (24–26). These concordances strengthen confidence that BALF-based genotyping reflects true tumor biology rather than technical artifact and may serve as a complementary tool when tissue sampling is limited or inconclusive.

Several limitations should be acknowledged. First, this was a single-center retrospective study with a relatively modest sample size (n = 100), which may limit generalizability and introduce potential selection bias. External validation in larger, multicenter prospective cohorts is warranted to confirm the robustness of these findings. Second, the benign pulmonary control group consisted of patients with non-malignant pulmonary conditions rather than healthy individuals. Although this design reflects real-world diagnostic practice, underlying inflammatory or infectious processes may influence cytokine levels and immune-cell distributions in BALF, thereby potentially confounding comparisons. Third, the cross-sectional design precluded evaluation of the temporal dynamics of BALF biomarkers. Longitudinal sampling was not performed; therefore, dynamic changes during treatment or disease progression could not be assessed. Fourth, although BALF ctDNA demonstrated higher detection rates and concordance compared with plasma, the targeted sequencing panel and sequencing depth may have limited the detection of low-frequency variants. Additionally, technical variability in BALF collection, processing, and handling may have influenced ctDNA yield and quantification. Fifth, immune profiling was restricted to selected immune subpopulations and did not comprehensively characterize the broader tumor immune microenvironment. Functional assays were not performed to validate the underlying biological mechanisms. Emerging evidence suggests that immune-cell metabolic reprogramming—including glucose metabolism–driven T-cell dysfunction (27) and glutamine metabolism–dependent immune regulation (28), plays a critical role in shaping the tumor microenvironment. These metabolic–immune interactions were not evaluated in the present study and warrant further investigation. Finally, although multivariable analysis identified independent prognostic factors, residual confounding cannot be entirely excluded. The limited sample size may also affect the stability and precision of Cox regression estimates.

Overall, our findings support BALF as a tumor-proximal, information-rich biospecimen that captures convergent signals from protein markers, inflammatory networks, tumor genomics, and local immunity. The integration of these dimensions highlights the potential clinical utility of BALF for minimally invasive diagnosis, risk stratification, and therapeutic guidance. Future multicenter studies with harmonized pre-analytical protocols, external validation, and longitudinal sampling across treatment courses are warranted to define standardized BALF workflows and confirm clinical benefit at scale.

## Conclusion

Multidimensional biomarker analysis of bronchoalveolar lavage fluid (BALF) provides a sensitive and minimally invasive approach for lung cancer evaluation. Elevated tumor markers, inflammatory imbalance, particularly reflected by the IL-6/IL-10 ratio, enhanced ctDNA detection, and distinct immune-cell patterns in BALF collectively improve diagnostic discrimination and prognostic assessment. Integrating these parameters using conventional statistical approaches underscores the value of BALF as a comprehensive liquid biopsy for precision evaluation and risk stratification in lung cancer.

## Data Availability

The raw data supporting the conclusions of this article will be made available by the authors, without undue reservation.
