# Supplementary material for: Multidimensional biomarker profiling of bronchoalveolar lavage fluid for diagnostic and prognostic evaluation of lung cancer: a retrospective observational study
Source: Front Med (Lausanne). 2026 Mar 27;13:1774382. doi: 10.3389/fmed.2026.1774382 (PMC13066139; doi:10.3389/fmed.2026.1774382)
Supplement: Supplementary file 1 [file Table_1.docx]

**Supplementary Table S1.** Immune cell subpopulations in BALF.Values are presented as mean ± SD.

| **Cell subpopulation** | **Lung cancer (n = 65)** | **Benign control (n = 35)** | **P value** |
| --- | --- | --- | --- |
| CD3⁺ T cells (%) | 42.5 ± 11.7 | 49.8 ± 13.5 | 0.006 |
| CD4⁺ T cells (%) | 24.3 ± 8.9 | 20.6 ± 7.4 | 0.040 |
| CD8⁺ T cells (%) | 16.8 ± 5.7 | 28.5 ± 8.2 | <0.001 |
| CD4⁺/CD8⁺ ratio | 1.45 ± 0.54 | 0.72 ± 0.28 | <0.001 |
| CD4⁺Foxp3⁺ Treg (%) | 10.7 ± 3.8 | 4.2 ± 1.6 | <0.001 |
| CD8⁺/Treg ratio | 1.6 ± 0.7 | 6.8 ± 2.1 | <0.001 |
| CD68⁺CD80⁺ M1 (%) | 27.3 ± 8.2 | 45.2 ± 11.7 | <0.001 |
| CD68⁺CD206⁺ M2 (%) | 58.4 ± 10.6 | 32.7 ± 9.4 | <0.001 |
| M1/M2 ratio | 0.47 ± 0.18 | 1.38 ± 0.52 | <0.001 |
| CD11b⁺CD33⁺HLA-DR⁻ MDSC (%) | 5.8 ± 2.7 | 2.3 ± 1.4 | 0.002 |
